# Microstrip Patch Strain Sensor Miniaturization Using Sierpinski Curve Fractal Geometry

**DOI:** 10.3390/s19183989

**Published:** 2019-09-15

**Authors:** Michal Herbko, Przemyslaw Lopato

**Affiliations:** Department of Electrical and Computer Engineering, West Pomeranian University of Technology, Szczecin, ul. Sikorskigo 37, 70-313 Szczecin, Poland

**Keywords:** strain sensor, microstrip sensor, microstrip antenna, fractal, microwave technique, SHM, NDT

## Abstract

In this paper miniaturization of a microstrip patch strain sensor (MPSS) using fractal geometry was proposed and analyzed. For this purpose, the transducer of Sierpinski curve geometry was utilized and compared with the most commonly utilized rectangular resonator-based one. Both sensors were designed for the same resonant frequency value (2.725 GHz). This fact allows analysis of the influence of the patch (resonator) shape and size on the resonant frequency shift. This is very important as the sensors with the same resonator shape but designed on various operating frequencies have various resonant frequency shifts. Simulation and experimental analysis for all sensors were carried out. A good convergence between results of simulation and measurements was achieved. The obtained results proved the possibility of microstrip strain sensor dimensions reduction using Sierpinski curve fractal geometry. Additionally, an influence of microstrip line deformation for proposed sensors was studied.

## 1. Introduction

Safety assurance is very important task in case of civil structures. For many years, civil constructions were evaluated using periodic inspections. However, these have to be conducted by qualified personnel. Furthermore, there is a lack of information about the structure condition during or just after extreme weather anomalies. For this reason, Structural Health Monitoring (SHM) systems are increasingly utilized [1,2]. SHM technique enables to increase structure safety by evaluation of construction condition in real time. A typical SHM system consists of sensors network, central data acquisition node, and algorithms. Various types of parameters are measured by the SHM sensor network from which the strain is crucial for assessment of structure state. 

A number of devices are utilized to measure strain. In 1938, Edward E. Simmons and Arthur C. Ruge invented and commercialized the resistant strain gauge [3]. Even though the invention is almost one hundred years old, it is still the most frequently used. So far, different types of resistive strain gauges have been designed such as wire, frame, and foil based ones. The resistance strain gauges use the phenomenon of changing the electrical resistance of the conductor due to the change in its length and cross-sectional area. The value of wire electrical resistance increases when it is stretched. Both the shape of the resistive mesh and the type of conductive material affect a transducer’s sensitivity. The change in strain gauge resistance is usually determined by a Wheatstone bridge or potentiometer circuit. The single strain gauge can evaluate the stress level in only one direction. When the force direction is unknown, strain is measured using rosette strain gauges.

Another device for strain measurement is a capacitive strain sensor. Its deformation influences the capacitance value. These sensors are especially useful for measuring stresses in applications with high elasticity. They consume about ten times less energy than resistive sensors, which is very important in case of wireless systems [4]. Thus, capacitive strain sensors can be applied in the measurement of skin or tire strain [4,5,6]. Until now, two types of capacitive strain sensors have been presented. The parallel-plate strain sensor is assembled by sandwiching a dielectric layer between two electrodes [6]. Whereas, the second type has in-plane electrodes parallel to their substrate [4,5]. Sensors with in-plane electrodes proved higher sensitivity, sensing linearity and lower hysteresis than parallel-plate-type capacitive strain sensors [5].

Strain measurement can also be performed using piezoresistive strain sensors, which are based on semiconductors. Strain deforms the semiconductor crystal lattice, which influences energy bands. As a result, the resistance value of the semiconductor element changes [7]. The prime benefit of the piezoresistive strain sensors is a considerably higher sensitivity compared with the resistant strain gauges. Nevertheless, they are very sensitive to temperature variance [8].

Piezoelectric sensors are also utilized in SHM systems for the strain measurement. The change in applied stress causes an electrical potential inside the amorphous crystals or selected polymers. This kind of sensor has a very high sensitivity. Thus, it has found application in dynamic force measurement [9,10]. It should be noted that the accumulated electric charge expires after a certain time of constant stress. Therefore, piezoelectric sensors are not suitable for static strain measurements [10].

Stress evaluation using magnetic sensors is carried out by measuring the stress dependent magnetic properties of the material, as opposed to sensors in which stress is measured through sensor deformation. Therefore, they can only be utilized to monitor stresses in ferromagnetic materials. The advantage of this solution is the ability to assess the stress level of the old structure, without new structure reference measurements [11,12]. Geometrical dimensions of magnetic sensors are definitely larger compared with other types of sensors.

Stress measurement can also be performed by many types of optical sensors [13]. Notwithstanding, the most popular sensor utilized in this application is the one based on Fiber Bragg Grating (FBG). FBG is introduced during the optical fiber production process by modulation of the core refractive index. FBG reflects the light differently from the rest of the fiber, as specific wavelengths are reflected. The dimensions of FBG are changed when the optical fiber is deformed. This causes changes of the refractive index and consequently wavelength of reflected light. The most important advantages of fiber optic sensors are immunity to electromagnetic interference, low weight, and high sensitivity. FBG strain sensors enable the placement of multiple sensors on one fiber optic cable. Nevertheless, they are expensive to build and then maintain.

In the last decade, the idea of using microstrip antennas for strain assessment was introduced. The works on improving this kind of transducer are conducted by many scientists [14,15,16,17,18,19,20,21,22,23,24,25,26,27,28]. The critical element of the microstrip antenna system was the Vector Network Analyzers (VNA). So far, the VNAs have been very expensive. Notwithstanding, low-cost VNAs (Pocket VNA, miniVNA Tiny) have appeared in recent years. Thus, it is possible to implement this type of systems in real structures. In addition, other cheap wire-based antenna interrogation mechanisms have been created, such as Frequency Modulated Continue Wave (FMCW) interrogator [29]. Moreover, the antenna sensor has the possibility of wireless interrogation—even without using batteries [30,31,32,33,34]. This property distinguishes this type of sensor from the others and provides the possibility to perform measurements in severe conditions or on rotating elements. As reported in literature, wireless measurement is most often used to measure strain and temperature. The main problem of wireless measurement is that signals received by the wireless interrogator consist of signal backscattered by the microstrip sensor and background clutter which creates the “self jamming” problem [30]. Up to now, various wireless measurement methods were described [30,31,32,33,34]. Both sensor and interrogated element are passive structures [31,32]. Thus they can be used to measure very high temperature (over 400°C). Other wireless sensors are based on the Radio Frequency Identification (RFID) technology [33]. The RFID-based interrogation, is appropriate only for static strain measurement due to its low interrogation speed. For wireless dynamic deformation measurement FMCW radar was developed [30]. An interesting way to solve the self-jamming problem is a sensor node consisting of two microstrip antennas. The first patch antenna is used as the transmitting/receiving (Tx/Rx) device. The second microstrip antenna is serving as the temperature sensor. A transmission line, connecting both microstrip antennas, delays the signal reflected from the sensing element and thus separates it from the background clutter [34]. The microstrip patch sensor is mechanically fixed to the evaluated element. Thus, its deformation causes a change in the patch geometry and current density distribution. This affects the resonant frequencies of the patch resonator. Strain evaluation using the microstrip antenna sensor is based on reflection coefficient *S*_11_ measurement in the frequency domain. The shift of resonant frequency Δ*f*_r_ is defined as follows:

Δ*f*_r_ = *f*_rload_ − *f*_r0MPa_ (1)

where *f*_rload_ is the resonant frequency for setup with external mechanical load, and *f*_r0MPa_ is the resonant frequency for setup without load.

Until now, various aspects of microstrip strain transducers were studied. Both wired and wireless microstrip strain sensors were tested [14,21]. The curvature effect during bending of the examined element was also considered [24]. The most commonly used in case of deformation evaluation was the rectangular-shaped patch [11,16,17,18,22,23,24,27,28]. Furthermore, circular [14,15,21,25] and other shapes [19,26] of resonators were utilized. The comparison of the resonant frequency shifts for studied sensors is shown in Table 1. The most sensitive to strain (the highest Δ*f*_r_ for defined strain level) are rectangular and circular patches (first resonant frequency). The last column of Table 1 contains the information about type of deformation, because the curvature caused by bending process also affects the sensitivity [24]. 

In most of the works just one resonant frequency was analyzed. Unfortunately, in this case the strain measurement cannot be performed when the exact angle of external force is unknown. This is due to different resonance frequency shifts caused by different force directions—non isotropic sensitivity. For some directions of mechanical excitation, the sensitivity can be even close to zero. This problem was solved by monitoring of two resonant frequencies, each associated with different distributions of current density in the resonator [14,23]. It results in various directional characteristics of the deformation for both resonances. In this case rectangular [23] and circular patch sensors were utilized. For the rectangular one, at the first resonance, the current is parallel to the main resonator axis whereas for second one is perpendicular. However, in the case of a circular microstrip sensor at the first resonance the current is also parallel to the main axis of the patch, while at the second resonance the current density distribution is more multi-directional. The strain measurement performed by two resonant frequencies monitoring enables the examination of direction and value of stress.

The maximum dimensions of microstrip strain sensors are in the range of 0.5λ to 6λ depending on configuration (where λ is wavelength). In some stress measurement applications, it is necessary to use smaller sensors. This is very important in cases where there is very little space to mount the transducer or when the local deformation gradient must be measured. This fact caused the need for miniaturization of sensors. In the telecommunication applications, where the frequency bands are strictly defined, the following aspects are considered to miniaturize microstrip antennas:

- use of high permittivity laminates,
- introduction of various slots in the ground plane,
- change and optimization of patch shape.

For deformation measurements, the microstrip patch sensor can be designed for higher operating frequencies. The dependence between sensitivity and sensor’s resonant frequency for rectangular patch was examined in [28]. The studied sensor had operating frequencies from 0.5 to 15 GHz. Resonance frequency shifts were from 0.5 to 15.1 MHz. Moreover, the same relationship was designated for the circular resonator [25]. The tested sensors were designed for operating frequencies from 0.5 to 5 GHz. Resonance frequency shifts from 0.5 to 4.8 MHz were obtained. Microstrip transducers designed for higher resonant frequencies have higher sensitivity and smaller size. So it seems that designing sensors for high operating frequencies is the most beneficial. However, the prices of Vector Network Analyzers depend on their maximum measurement frequency, thus utilization of very high frequencies becomes economically unjustified. Moreover, the sensitivity of microstrip patch sensors made on various permittivity substrates was evaluated. Similar frequency changes were obtained for all considered laminate electrical permittivities, but the patch radius length for *ε*_r_ = 2.2 was 23.7 mm, and for *ε*_r_ = 13.2 it was equal to 9.677 mm [25]. Thus, this method of miniaturization is very beneficial because it enables to obtain the same sensitivity and simultaneously to miniaturize the transducer.

This article presents another method of MPSS miniaturization based on selection and change of patch shape. In case of telecommunication applications patches were modified by loading the edges of the patch with inductive elements [35]. Moreover, fractal patches geometries are also often used to miniaturize the microstrip antennas [36,37,38]. In this paper Sierpinski curve fractal geometry was applied. According to the authors’ knowledge, this is the first application of fractal geometry in microstrip strain/stress sensors. It will enable sensor diminution without the use of expensive microwave laminates or may be an additional option using a high electrical permittivity laminate and fractal geometry to achieve an even bigger reduction in the deformation measurement area. The proposed fractal geometry in case of three iterations was studied and compared with a rectangular-shaped MPSS sensor designed for the same operating frequency. Thus, only the influence of the patch shape on sensor sensitivity was studied. 

## 2. Sensor Design

**Figure 1:**
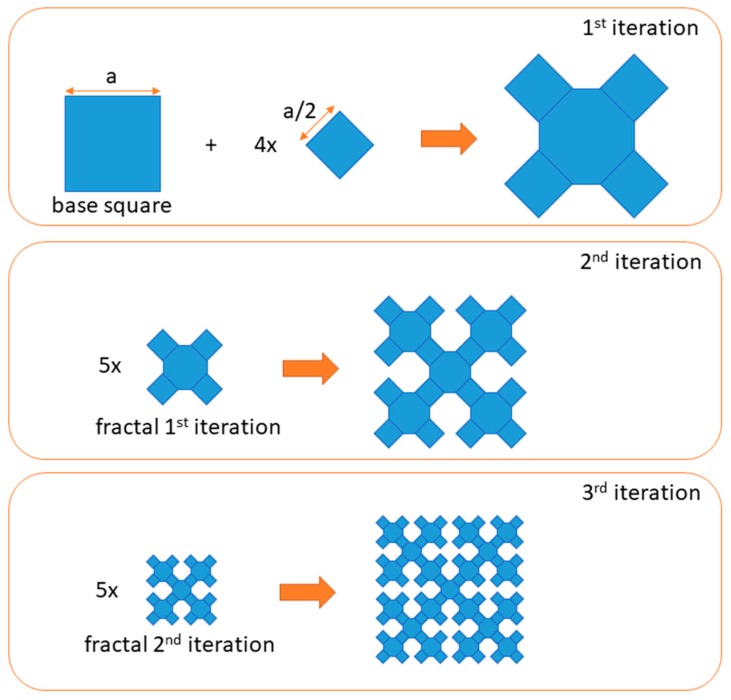
Construction method of Sierpinski curve fractal.

**Figure 2:**
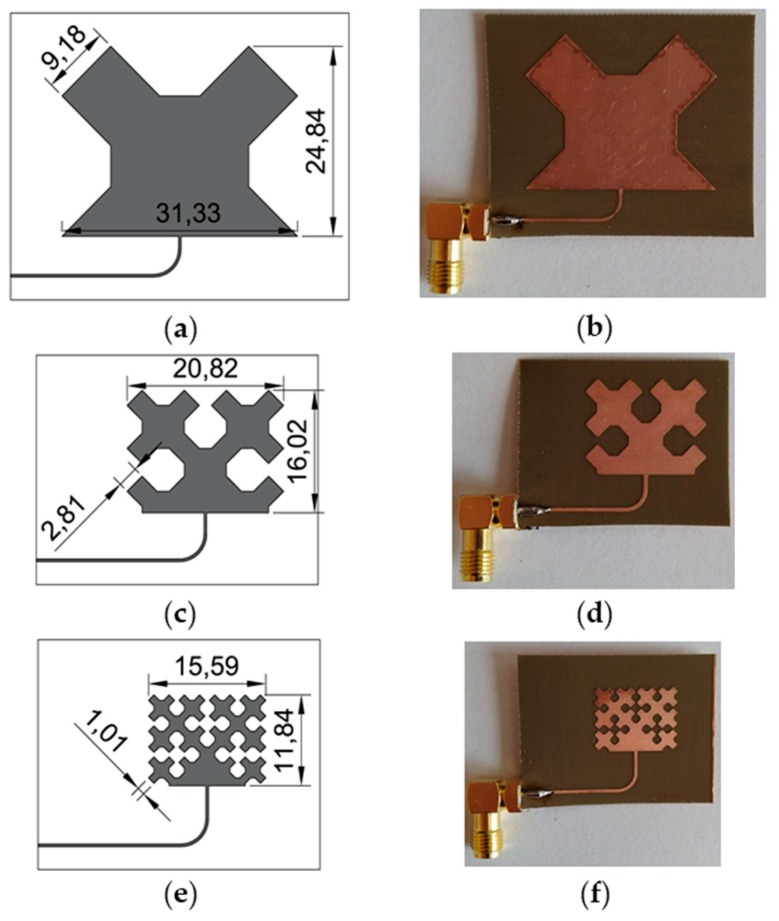
Designed Sierpinski curve based fractal strain sensors; (**a**) view and dimensions (in mm) of first iteration fractal sensor; (**b**) photo of first iteration fractal sensor; (**c**) view and dimensions (in mm) of second iteration fractal sensor; (**d**) photo of manufactured second iteration fractal sensor; (**e**) view and dimensions (in mm) of third iteration fractal sensor; (**f**) manufactured third iteration fractal sensor.

Until now, various fractal microstrip antennas were presented in the literature. Notwithstanding, fractal patches were not investigated in strain measurement applications yet. Many various fractals were created and even more reports on the use of fractal geometry in telecommunications applications were published. This is due to the fractal geometry modifications and various feed methods. One of the most known fractal creators was Polish mathematician Wacław Sierpiński. He invented three well-known fractals, which were later named Sierpinski fractals (the Sierpinski gasket, the Sierpinski carpet, and the Sierpinski curve). So far, microstrip antennas using these three fractal geometries were built and tested [36,37,38]. In this work, it was decided to manufacture and examine the patches based on the geometry of the Sierpinski curve in the strain assessment application. This shape enables significant reduction of the resonator size [36]. The construction method of Sierpinski curve fractal is presented in Figure 1. Obtained fractal geometry, was modified in order to feed it by microstrip line as shown in Figure 2. The sensors were designed on FR4 laminate (*ε*_r_ = 4.4, tanδ = 0.02). 

**Figure 3:**
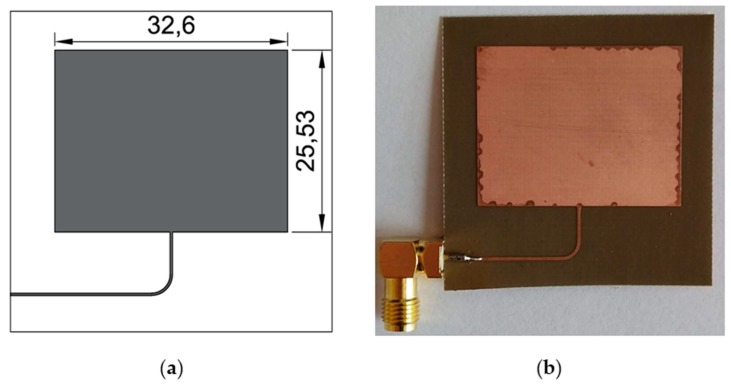
Rectangular microstrip strain sensor; (**a**) view and dimensions (in mm) of rectangular microstrip strain sensor; (**b**) photo of manufactured rectangular microstrip patch strain sensor (MPSS).

The same patch modification was carried out in [36]. All sensors were designed for the same operating frequency (2.725 GHz). This value was selected as a compromise between high sensitivity (high resonant frequency) and the possibility of using low-cost VNA (up to 3 GHz) and low-cost laminates. Three designed iterations of Sierpinski curve fractal strain sensor are shown in Figure 2. In order to compare proposed sensor with standard and commonly utilized one, a rectangular microstrip strain sensor was designed using the formulas presented in [39]. Dimensions and a photo of the fabricated rectangular microstrip transducer are shown in Figure 3.

## 3. Numerical Analysis

**Figure 4:**
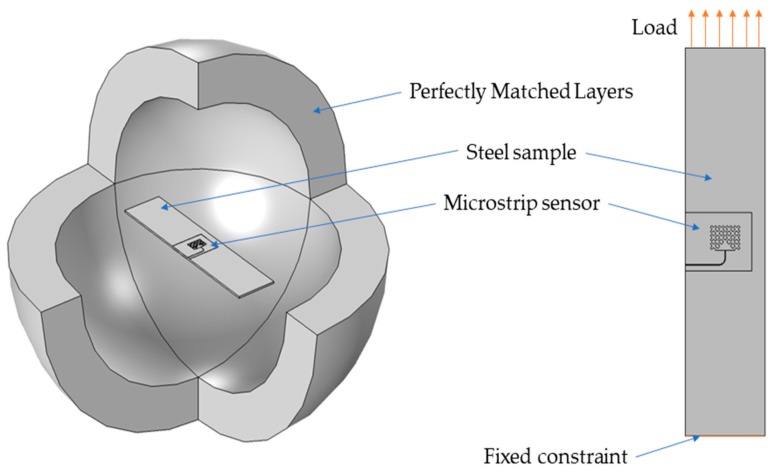
Finite Element Method (FEM) numerical model.

**Figure 5:**
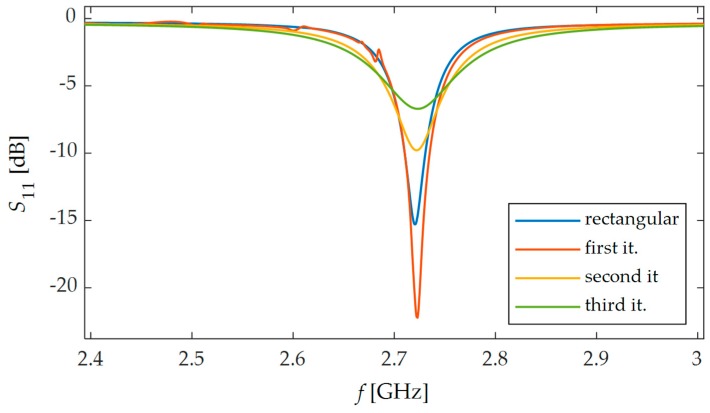
Calculated reflection coefficient characteristics *S*_11_ for proposed microstrip transducers.

**Figure 6:**
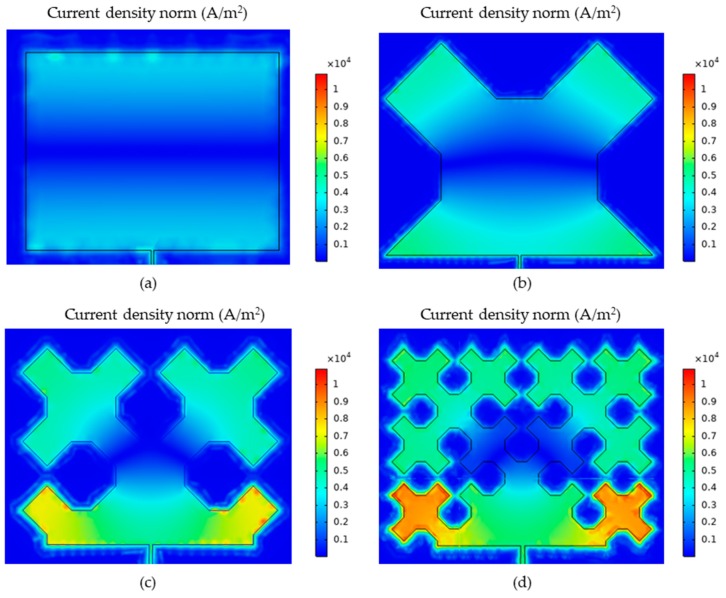
Current density distribution for resonant frequency; (**a**) rectangular patch; (**b**) Sierpinski curve fractal first iteration; (**c**) Sierpinski curve fractal second iteration; (**d**) Sierpinski curve fractal third iteration.

**Figure 7:**
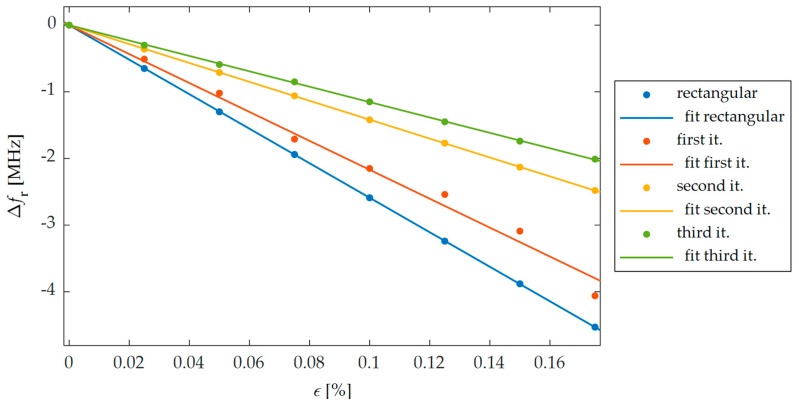
Dependencies between strain *ε* and shifts of resonant frequency Δ*f*_r_ for proposed sensors obtained in numerical analysis.

FEM (Finite Element Method) model for study of proposed sensors in Comsol Multiphysics environment was developed. This numerical model is shown in Figure 4. The sensor was fixed to the S355J2+N construction steel sample. Thus, the designed transducers were evaluated using a material, which is widely used in civil structures. Firstly, the Solid Mechanics module was utilized to simulate deformation of the sample. Mechanical loading of the sample causes simultaneous deformation of the MPSS. As a result, the current density distribution in the patch changes as well as the frequency domain reflection coefficient (*S*_11_) characteristics. The transducers resonant frequency was determined based on the received reflection coefficient characteristics calculated using the RF module. Reflection coefficient characteristics determined using numerical analysis are presented in Figure 5. As it can be seen, all microstrip patch sensors have the same operating frequencies. The current density distributions in the resonators at the resonant frequency are shown in Figure 6. For the rectangular patch and the first iteration of Sierpinski curve fractal resonator, the current is parallel to the main axis of the patch. However, for higher iterations of the fractal, the current distribution is more multidirectional. For this reason, bigger shifts of resonant frequencies were obtained for a rectangular resonator and for lower fractal iterations (Figure 7).

**Figure 8:**
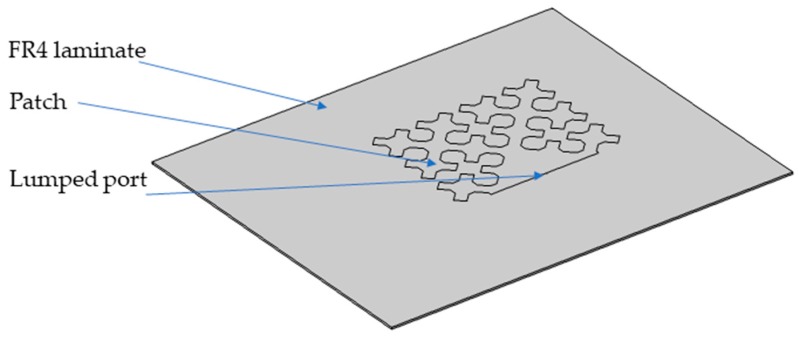
Fractal MPSS feed without transmission line.

The proposed sensor is fed by microstrip transmission line. Thus mechanical strain applied on the steel sample, causes dimensions change of both patch and microstrip line. For this reason, the behavior of proposed sensor was also numerically studied without the microstrip line; the excitation port was placed directly on the patch (the position of patch feed is shown in Figure 8). The results obtained are shown in Table 2. As one can see, the effect of microstrip line deformation is below 5%. This analysis showed that the deformation of the microstrip line has little effect on the reading of the real local deformation level under the patch.

## 4. Experimental Analysis

**Figure 9:**
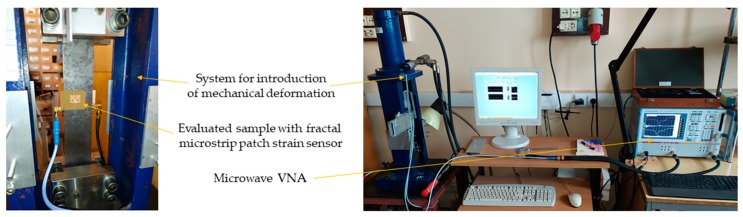
Photo of measuring system setup.

**Figure 10:**
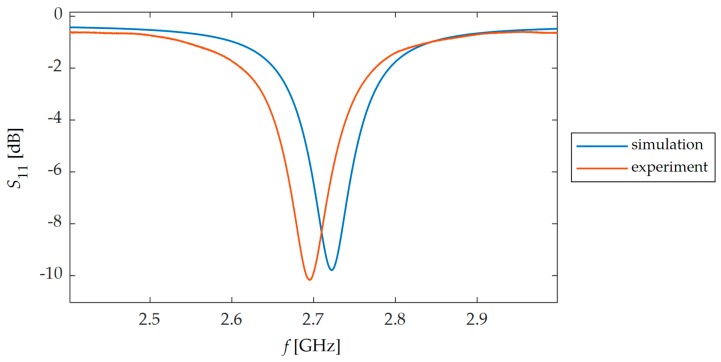
Calculated and measured reflection coefficient characteristics *S*_11_ for second iteration of Sierpinski curve fractal.

**Figure 11:**
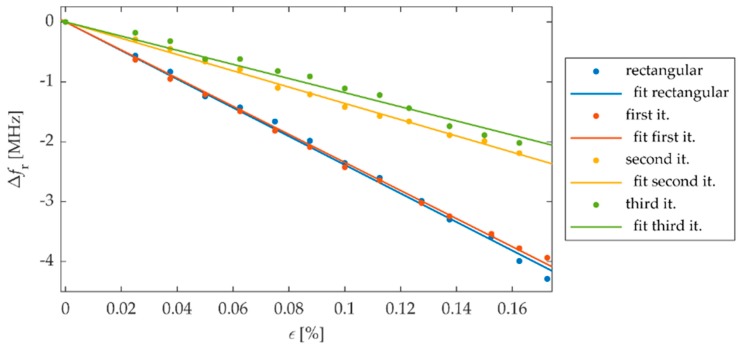
Dependencies between strain *ε* and shifts of resonant frequency Δ*f*_r_ for proposed sensors received in measurement.

In this section, experimental verification of proposed and simulated transducers was carried out. For this purpose, four microstrip patch sensors were manufactured using photolithographic process. Designs and photos of their implementation are presented in Figure 2 and Figure 3. These sensors were attached with cyanoacrylate adhesive to the steel samples. This adhesive connection enables the transmissions of sample strain to studied MPSS. The steel sample was deformed using hydraulic system for introduction of mechanical deformation. Afterwards, for the deformed sensor Rohde&Schwarz ZVB20 Vector Network Analyzer (VNA) was utilized for reflection coefficient *S*_11_ acquisition. Measurement was conducted in the 2.4–3 GHz frequency range with 0.25 MHz step. The measurement setup is presented in Figure 9. Measured and calculated reflection coefficient characteristics for exemplary sensor are presented in Figure 10. Comparing the results presented in Figure 7 and Figure 11, one can observe that a good convergence between simulations results and experimental verification results was obtained. The largest resonance frequency shifts were obtained for the rectangular microstrip patch sensor and the first iteration of the Sierpinski curve fractal. Whereas higher iteration of fractal MPSS is less sensitive on strain.

## 5. Conclusions

In this paper, the miniaturization of the microstrip strain sensor using a specific patch shape was evaluated. For this purpose, Sierpinski curve based fractal geometry was utilized and sensitivity test was carried out for three iterations of this fractal. The main criterion for choosing this fractal geometry was the possibility of significantly reducing the study area. Obviously, similar sized sensors can be obtained using other fractal geometries. Nevertheless, various shapes of patch have different sensitivities. For this reason, in further work other fractal geometries will be considered to select the best solution. In addition, a comparative study with a rectangular patch was made. Comparison of received results was shown in Table 3. First of all, a good convergence between the results of numerical and experimental analysis was obtained. Small differences between simulation results and measurements are caused by inaccuracies in the production of resonators, lack of knowledge regarding the exact values of the electromagnetic and mechanical parameters of the laminate (these values were obtained from producer, not measured for specific samples), and approximation errors. Due to the high stiffness of the adhesive and the thin laminate (h = 0.18 mm), the shear lag effect does not much affect the results. This effect was not taken into account during simulations. Furthermore, the dimensions of the sensor were significantly reduced by using a fractal patch. Unfortunately, for smaller fractal resonators, there is a smaller resonant frequency shift and in consequence lower sensitivity. An area of the sensor geometry that is four times smaller caused only twice-lower sensitivity. Thus, a better method for miniaturization of microstrip strain sensor is the utilization of the high permittivity laminate. The use of fractal patch can be used in combination with high electric permittivity laminate in the case when sensitivity requirements are lower and stress measurement is required on a very small area. Studies presented in the literature show that the largest resonant frequencies shift for stresses parallel to the main patch axis are when the current is parallel to the main patch axis. On the other hand, multidirectional current distribution in the patch allows obtaining higher sensitivity for other strain angles (Table 1). For the third iteration, the current density distribution is the most multidirectional. So this sensor may be more sensitive on strain in various directions.

Repeatability and strain measurement range are strongly dependent on the laminate and the adhesive connection mechanical properties. For this reason, thin and elastic laminates should be utilized in this application, while adhesives should have similar properties to the ones utilized in resistant strain gauges or other strain evaluation techniques. In order to obtain high repeatability, both laminate and adhesive connection should be resistant to fatigue damage and have low temperature dependence. In case of layered laminates there is a risk of delamination due to high compressive forces (related to nonlinear region of the stress–strain curve). Such changes in laminate structure can noticeably affect repeatability of the sensor. The measurements and analysis were carried out in a linear (elastic) region of the stress–strain curve because this type of sensor was considered for measuring steel structures. Exceeding the yield point causes loss of integrity of the monitored structure, thus (because of safety issues) during such structure design process even at the highest load, the operating point must be within the linear range (with a wide safety margin).

## Figures and Tables

**Table 1:** Comparison of microstrip sensors.

Patch Shape	*f*_r_ [GHz]	Δ*f*_r ε=0.1%_ [MHz]	Type of Deformation
Rectangular patch feed by microstrip line [23]	17.7	14.3	Non-planar (bending)
Rectangular patch feed by microstrip line [28]	0.5–15	0.5–15.1	Planar
Circular patch feed by microstrip line— first resonant frequency [14]	2.5	2.3	Planar
Circular patch feed by microstrip line— second resonant frequency [14]	4.3	1.2	Planar
Circular patch feed by microstrip line [25]	0.5–5	0.5–4.8	Planar
Slotted patch feed by coaxial probe [19]	3.4	3.1	Non-planar (bending)

**Table 2:** Shift of resonant frequency for proposed sensors feed with microstrip line and without it.

Patch Shape	Δ*f*_r ε=0.1%_ [MHz]—Feed by Microstrip Line	Δ*f*_r ε=0.1%_ [MHz]—Feed without Microstrip Line
Rectangular	−2.59	−2.63
First iteration of Sierpinski curve fractal	−2.17	−2.13
Second iteration of Sierpinski curve fractal	−1.42	−1.45
Third iteration of Sierpinski curve fractal	−1.15	−1.2

**Table 3:** Comparison of the examined sensor.

Patch Shape	Resonator Size [mm^2^]	Δ*f*_r ε=0.1%_ [MHz]—Simulation	Δ*f*_r ε=0.1%_ [MHz]—Measurement
Rectangular	832.28	−2.59	−2.39
First iteration of Sierpinski curve fractal	778.24	−2.17	−2.35
Second iteration of Sierpinski curve fractal	333.54	−1.42	−1.36
Third iteration of Sierpinski curve fractal	184.59	−1.15	−1.18
